# Previously Uncharacterized Variants, OCF-E–OCF-J, of the Antifungal Occidiofungin Produced by *Burkholderia contaminans* MS14

**DOI:** 10.1021/acs.jnatprod.3c00777

**Published:** 2024-01-26

**Authors:** Nopakorn Hansanant, Kevin Cao, Abraham Tenorio, Thushinari Joseph, Min Ju, Noah McNally, Evangel Kummari, McKinley Williams, Andrew Cothrell, Andrew R. Buhrow, Ronald Shin, Ravi Orugunty, Leif Smith

**Affiliations:** † Department of Biology, Texas A&M University, College Station, Texas 77843, United States; ‡ Sano Chemicals Incorporated, Bryan, Texas 77803, United States; § Central Alabama High-Field NMR Facility, Structural Biology Shared Facility, Cancer Center, University of Alabama at Birmingham, Birmingham, Alabama 35294-1240, United States

## Abstract

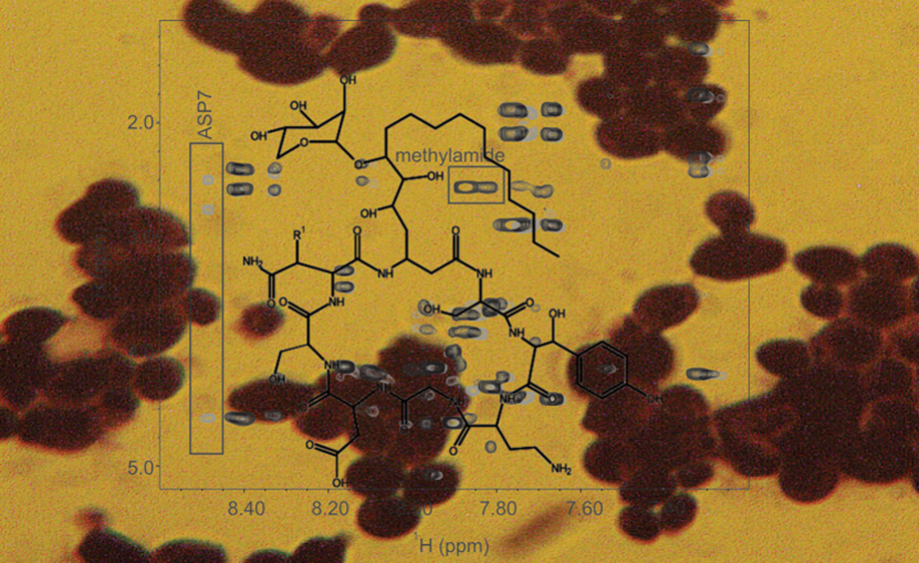

The rise of multidrug resistant fungal infections highlights the need to identify and develop novel antifungal agents. Occidiofungin is a nonribosomally synthesized glycolipopeptide that has a unique mechanism of action, disrupting actin-mediated functions and inducing cellular apoptosis. Antifungal activity has been observed in vitro against various fungal species, including multidrug resistant *Candida auris*, and in vivo efficacy has been demonstrated in a murine vulvovaginal candidiasis model. Occidiofungin, a cyclic glycolipopeptide, is composed of eight amino acids and in previous studies, an asparagine residue was assigned at position 7 (ASN7). In this study, new structural variants of occidiofungin have been characterized which have aspartic acid (ASP7), glutamine (GLN7), or glutamic acid (GLU7) at position 7. The side chain of the ASP7 variant contains the only terminal carboxylic acid in the peptide and provides a useful site for selective chemical modifications. Analogues were synthesized at the ASP7 position and tested for antifungal activity. These analogues were shown to be more active as compared to the ASP7 variant against a panel of *Candida* species. The naturally occurring variants of occidiofungin with a side chain containing a carboxylic acid at the seventh amino acid position can be used to develop semisynthetic analogues with enhanced therapeutic properties.

The global annual incidence of invasive fungal infections is estimated to be nearly two million cases.^1^ The rise of drug resistant fungal infections poses a serious health threat due to the lack of viable treatment options.^2−4^ Amphotericin B or a combination of amphotericin B with other antimicrobials is usually the last line of treatment option in most of these cases, but invasive fungal infections still have a mortality rate between 25%–45%.^5^ The emergence of *Candida auris* strains resistant to existing antifungal treatments is alarming, particularly due to outbreaks occurring in geriatric and healthcare facilities.^6,7^ The development of a new class of antifungals is therefore crucial for the treatment of emerging drug-resistant fungal infections.

Occidiofungins are potent antifungal compounds produced by the soil bacterium *Burkholderia contaminans* MS14.^8^ When stating occidiofungin, in singular, in this manuscript, we are referring to the structural class. Structurally, occidiofungin is a cyclic glycolipopeptide, that is produced nonribosomally. It is composed of eight amino acids (Figure 1). The biosynthesis of occidiofungin is controlled by the *ocf* gene cluster, which is a hybrid polyketide synthase (PKS) and nonribosomal peptide synthase (NRPS) pathway (Figure S1 of the Supporting Information). NRPS systems can have promiscuity in amino acid selection, and this has been observed in several bacterial systems.^9−11^ Several natural variants of occidiofungin have been previously identified.^8^ The NRPS OcfJ is predicted to incorporate either asparagine (ASN1) or β-hydroxyasparagine (BHN1) in the first amino acid position in the peptide.^8^ The *ocf* gene cluster encodes for two thioesterases; one located on the C-terminal thioesterase domain of OcfD, and another independently expressed thioesterase OcfN. Knocking out OcfN in *B. contaminans* MS14 resulted in a reduction of occidiofungin conformational variants and these variants of occidiofungin produced by this mutant had reduced antifungal activity.^12^ Occidiofungin has a β-hydroxytyrosine (BHY) at position four, which can be chlorinated by the halogenase OcfK.^8^ The extensive repertoire of occidiofungin variants may be important for the antifungal activity of *B. contaminans* MS14 in the bacterium’s natural environment where it is likely to encounter a wide array of different fungal species.

**Figure 1:**
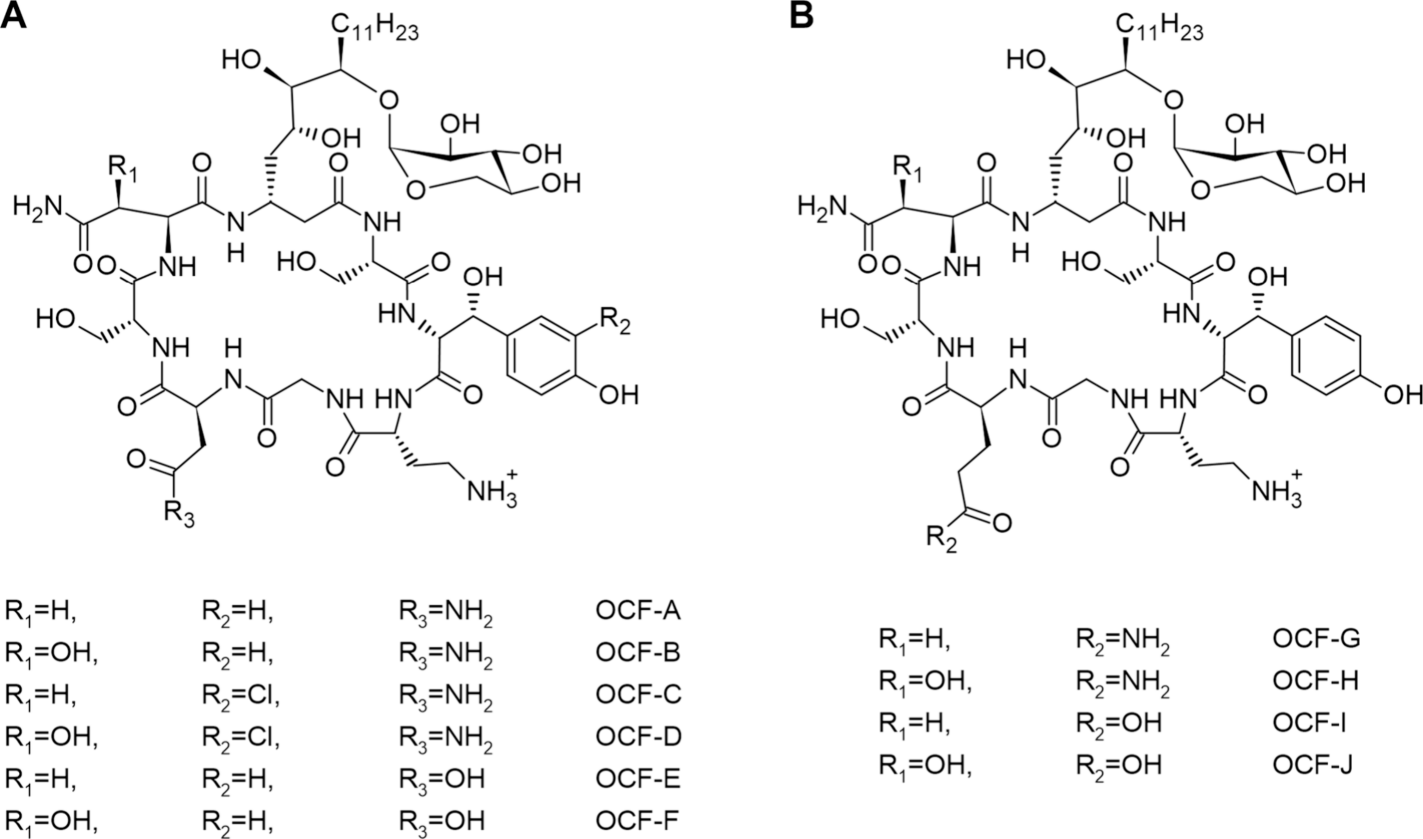
Representation of the covalent structures of the naturally occurring occidiofungin variants. In panel A, the OCF-A through OCF-F were derived from the asparagine and aspartic acid at position 7 variants. In panel B, the OCF-G through OCF-J were derived from the glutamine and aspartic acid variants.

The antifungal activity of occidiofungin has been demonstrated against a wide variety of fungal species, including multidrug resistant *Candida auris*.^13^ The disruption of higher order actin mediated activities, such as formation of actin cables, was determined to be the mechanism of action of occidiofungin,^13^ Occidiofungin does not interfere with F-actin polymerization or depolymerization,^13^ but was determined to inhibit actin-mediated cellular processes and trigger cellular apoptosis.^13,14^ This mechanism of action and minimal toxicity is unique compared to current antifungal agents, and makes occidiofungin a candidate for development into an antifungal agent.^13,15^ Toxicity and efficacy studies in murine models have demonstrated that occidiofungin has minimal toxicity and is effective in treating vulvovaginal candidiasis.^13^ Given the minimal toxicity and efficacy demonstrated by occidiofungin, the Food and Drug Administration has approved Phase 1 clinical trials for a drug product using occidiofungin to treat recurrent vulvovaginal candidiasis.^16^

Occidiofungin formulated in phosphate-buffered saline (PBS) containing 1.5% hydroxypropyl-β-cyclodextrin or in a liposomal formulation did not demonstrate efficacy in treating candidemia in a murine infection model.^17^ Liposomal encapsulated occidiofungin exhibited superior pharmacokinetic properties when compared to nonliposomal encapsulated occidiofungin. The *C*_max_ of encapsulated occidiofungin was observed to be one log higher than the minimal inhibitory concentration against *Candida* species.^17^ However, the improved pharmacokinetic properties were insufficient to reduce fungal load in the murine infection model. Occidiofungin has shown strong binding to serum proteins, and this was predicted to be the cause for the insufficient reduction of fungal load in a murine invasive infection study with the dose formulation tested.^17^ Mice and rat serums were shown to have a greater inhibitory effect on the antifungal activity of occidiofungin in vitro when compared to human serum and blood, thus suggesting that occidiofungin binds to serum proteins present primarily in rodent species with greater affinity than to human serum proteins.^17^ The serum binding studies open up possibilities for newer occidiofungin analogues that may have broader therapeutic applications. For example, analogues with reduced serum binding may hold promise toward developing an agent for treating invasive fungal infections.^17,18^

In addition to antifungal properties, occidiofungin has been shown to be cytotoxic to cancer cell lines and the parasite *Cryptosporidium parvum*. Occidiofungin has demonstrated in vitro cytotoxic activity against ovarian cancer OVAR8, astrocytoma brain cancer SW1088, and B-cell non-Hodgkin lymphoma cancer TOLEDO CRL-2631 cell lines.^15^ The TC_50_ of occidiofungin in these cell lines were approximately 8-fold lower compared to normal human dermal fibroblasts. Selective antiparasitic activity has also been demonstrated against the intracellular parasite *C. parvum* in an epithelial cell line.^19^ Occidiofungin was demonstrated to be stable following oral administration and was not absorbed through the gastrointestinal tract of mice.^19^ These properties make it a possible candidate for the selective treatment of intestinal parasites. The mechanism of action against cancer cell lines and *C. parvum* has not yet been investigated. Actin is vital for cellular function and is known to be highly conserved between eukaryotes,^20^ thus the intracellular target of occidiofungin is also likely to be actin in cancer cells and *C. parvum*. The development of occidiofungin analogues may be useful to broaden possible therapeutic applications which includes treatment of invasive fungal infections, parasitic infections, and cancer.

Occidiofungin is being developed as an antifungal agent, and concurrent experiments to improve the efficacy and therapeutic applications are being performed. Modifications and optimizations of methods toward the scaled-up production and purification of occidiofungin have revealed previously unidentified variants. These novel occidiofungin variants differ at the seventh residue; where aspartic acid (ASP7), glutamine (GLN7), or glutamic acid (GLU7) is present instead of asparagine (ASN7) (Figure 1). These new occidiofungin variants were isolated using high-performance liquid chromatography (HPLC) and ultrahigh-performance liquid chromatography (UHPLC) methods. Eluted peaks containing the occidiofungin variants were characterized through high resolution mass spectrometry (HR-MS) and nuclear magnetic resonance (NMR). Semisynthetic occidiofungin analogues were prepared using the ASP7 variants given that the side chain of this amino acid residue contains the sole carboxylic acid in occidiofungin. Most of these analogues demonstrated improved antifungal activity compared to the ASP7 variants in vitro. This indicates that converting the carboxylic acid at the seventh residue position into an amide improves the antifungal activity against yeast and that the region is amenable to a wide array of modifications.

## Results and Discussion

### Isolation and HR-MS Analyses of Distinct Variants

*B. contaminans* MS14 was grown in a modified minimal medium with asparagine as the sole carbon source. Preparative HPLC was used to isolate occidiofungin from a cell free extract (Figure S2). The occidiofungin fraction was analyzed by HR-MS and showed that the fraction contained new naturally occurring variants of occidiofungin. The observed masses were compared to theoretical masses of the elemental composition for each possible variant (Table 1). A mass error of up to 5 ppm was allowed for each assignment. The preparative HPLC fraction contained a variety of occidiofungin variants. In particular, the seventh amino acid position is predicted to have amino acid promiscuity with the substitution of either ASN7, GLN7, ASP7, or GLU7. The ASN7 and ASP7 occidiofungin variants were observed in either of the asparagine (ASN1) or β-hydroxyasparagine (BHN1) containing variants, while the GLN7 and GLU7 occidiofungin variants were only observed with BHN1 containing variants. Previously documented chlorinated β-hydroxytyrosine variants OCF-C and OCF-D were also not observed under these growth conditions. OCF-C and OCF-D variants were previously observed in cultures grown in potato dextrose broth and their absence could be attributed to culturing in minimal media.^8^ The absence of certain variants may be due to their low abundance or may reflect the need for certain culture conditions and components for their presence.

**Table 1:** Predicted Occidiofungin Variants and the Occidiofungin Variants Identified by HR-MS in the Preparative HPLC Fraction^a^

variant and elemental composition	residue 1	residue 4	residue 7	theoretical mass [M + H]^+^	observed mass [M + H]^+^	mass error (ppm)
OCF-A C_52_H_86_O_21_N_11_	ASN	BHY	ASN	1200.5994	1200.6027	2.7
OCF-B C_52_H_86_O_22_N_11_	BHN	BHY	ASN	1216.5943	1216.5974	2.5
OCF-C C_52_H_85_O_21_N_11_Cl	ASN	chlorinated BHY	ASN	1234.5610	not observed	
OCF-D C_52_H_85_O_22_N_11_Cl	BHN	chlorinated BHY	ASN	1250.5559	not observed	
OCF-E C_52_H_85_O_22_N_10_	ASN	BHY	ASP	1201.5839	1201.5874	2.9
OCF-F C_52_H_85_O_23_N_10_	BHN	BHY	ASP	1217.5784	1217.5824	3.3
OCF-G C_53_H_88_O_21_N_11_	ASN	BHY	GLN	1214.6156	not observed	
OCF-H C_53_H_88_O_22_N_11_	BHN	BHY	GLN	1230.6099	1230.6134	2.8
OCF-I C_53_H_87_O_22_N_10_	ASN	BHY	GLU	1215.5996	not observed	
OCF-J C_53_H_87_O_23_N_10_	BHN	BHY	GLU	1231.5946	1231.5983	3.0

a The amino acid heterogeneity for residue positions 1, 2, 4, and 7 contributed to the observed variants. OCF-C and OCF-D were observed in cultures grown in potato broth.^8^

The preparative HPLC fraction was further fractionated using UHPLC on an analytical column (Figure S3). The sample was separated into three main peaks which were collected separately and analyzed by HR-MS. The ASN7 variants eluted out in peak 1, containing OCF-A and OCF-B (Figure S3). The ASP7 variants eluted out in peak 2, containing OCF-E and OCF-F. The GLN7 variant OCF-H eluted out in peak 3, while the GLU7 variant (OCF-J) was not observed in these fractions. This was likely attributed to its overall low abundance in the preparative HPLC fraction. Peaks 1 and 2 contained both the ASN1 and BHN1 variants, while peak 3 contained only the BHN1 residue. The percent composition of each group of the variants in the preparative HPLC fraction was determined from the average of three distinct UHPLC runs. Peak 1 was the most abundant at 66.4% relative composition, peak 2 was 26.0% relative composition and peak 3 was 7.6% relative composition.

### Confirmation of the Amino Acid Promiscuity at Position 7

The preparative HPLC fraction, which contains the ASN7, ASP7, GLN7, and GLU7 variants was analyzed using 2D NMR TOCSY and NOESY experiments (Figures S4 and S5 and Table 2). A complete sequential walk could be made from ASN1/BHN1 to serine at position 8 (SER8) and from SER8 to ASN1/BHN1 (Figure S5). The ASP7, ASN7, and GLN7 spin systems were identified in the sample (Figures S4 and S5). NOEs were also observed from the H^α^ and H^β^ of the ASP spin system to H^N^ of SER8, and from H^α^ of glycine at position 6 (GLY6) to the H^N^ of the ASP spin system. NOEs were observed from H^N^ of GLY6 to H^N^ of ASN7 and from H^N^ of ASN7 to H^N^ of SER8 (Figure S5). The NMR analyses confirm the presence of the ASP residue at position 7 (ASP7) and not at any other position in the peptide. The HR-MS data identified the presence of the GLN7 and GLU7 variants (Table 2). Analyses of the 2D NMR data revealed the GLN7 spin system with an H^N^ shift of 8.30 and with the corresponding alpha, beta, epsilon, and gamma proton chemical shifts of a GLN (Table 2). NOEs were seen from H^α^ and H^β^ of the assigned GLN7 spin system to H^N^ of SER8. A spin system for GLU7 was not observed in the NMR data set. Given that it was not observed in the HR-MS data acquired on the three UHPLC fractions and given the weak MS intensity observed in the preparative HPLC fraction, it is expected to be a low abundant product. Further, the confirmation for the promiscuity at position seven comes from the NMR analysis of the isolated ASP7 variants OCF-E and OCF-F (Table S1). The spin systems for both the ASN1 and BHN1 variants were distinguishable and are responsible for the duplication of the other amino acid spin systems (Figure S6). An overlay of these assignments was made to the spin systems of the isolated preparative chromatography fraction shown in Figure S4 and confirmed that the assignment of the ASP7 spin systems was correct.

**Table 2:** ^1^H NMR Chemical Shift Values for the Preparative HPLC Fraction of Occidiofungin in Dimethyl Sulfoxide (DMSO-d6) at 25°C

amino acid position	H^N^	H^α^	H^β^	other protons
ASN1	8.09	4.52	2.52, 2.35	γ-NH2:7.26, 6.86
BHN1	7.90	4.63	4.02	γ-NH2:7.30, 6.93 β-OH: 5.70
NAA2	7.53			C2-CH2:2.32, 2.40 C3-CH: 4.15, C4-CH2:1.76, 1.35, C5-CH: 3.51, C5-OH: 4.17, C6-CH: 3.05, C6-OH:4.08, C7-CH: 3.75, C8-CH: 1.36, C9-C17-CH2:1.31, C18-CH3:0.86
	7.30			C2-CH2:2.32, 2.40 C3-CH: 4.15, C4-CH2:1.76, 1.35, C5-CH: 3.51, C5-OH: 4.17, C6-CH: 3.05, C6-OH:4.08, C7-CH: 3.75, C8-CH: 1.36, C9-C17-CH2:1.31, C18-CH3:0.86
	7.28			C2-CH2:2.32, 2.40 C3-CH: 4.15, C4-CH2:1.76, 1.35, C5-CH: 3.51, C5-OH: 4.17, C6-CH: 3.05, C6-OH:4.08, C7-CH: 3.75, C8-CH: 1.36, C9-C17-CH2:1.31, C18-CH3:0.86
				Xylose: C1-CH2:4.18, C2-CH: 2.99, C2-OH: 4.94 C3-CH:3:10, C3-OH: 4.94, C4-CH:3.30, C4-OH:4.93, C5-CH2:3.72,3.06
SER3	8.15	4.14	3.42, 3.29	β-OH: 4.96
	8.07	4.22	3.42, 3.34	β-OH: 4.97
BHY4	8.09	4.18	5.07	β-OH: 5.77, OH: 9.36, C2&C6-CH:7.15, C3&C5-CH: 6.68
DABA5	7.66	4.41	2.11, 1.90	γ-H: 2.92, NH2:7.73
GLY6	7.98	3.79, 3.68		
	7.85	3.84, 3.68		
	7.70	3.92, 3.61		
ASN7	8.41	4.59	2.58, 2.40	γ-NH2:7.41, 6.94
	8.32	4.56	2.59, 2.43	γ-NH2:7.41, 6.94
ASP7	8.47	4.58	2.74, 2.48	
GLN7	8.30	4.25	1.88, 1.73	ε.-NH2:7.15.14, 6.67, C3-CH2:2.12
SER8	7.85	4.33	3.59	β-OH: 4.83
	7.80	4.34	3.61	β-OH: 4.82
	7.77	4.22	3.60	β-OH: 4.82

### Assignment of Absolute Stereochemistry

To establish the absolute configuration of the amino acids comprising OCF, hydrolysis followed by derivatization with Marfey’s reagent was employed.^21^ The data showed the presence of Gly, l-Asp (for l-Asn), d-DABA and both l-Ser and d-Ser (Figure S7). In a previous study, InterProScan and NRPS-PKS web-based software predictions described the domains for each NRPS module.^8^ The modules for BHY4, DABA5, and Ser8 were predicted to all contain an epimerase domain. Taking Marfey’s derivatization and the software prediction data into consideration, the amino acids at positions 5 and 8 are d-DABA and d-Ser. There are no amino acid standards for the novel amino acid (NAA) or the BHY residues at positions 2 and 4. Given the presence of the epimerase domain in the module for BHY, d-BHY configuration was assigned. This assignment is further supported by the small coupling constant observed between H-2_BHY_ and H-3_BHY_ (∼3.0 Hz) supporting anti (2R, 3R) relative configuration.^22−24^ The absolute configuration of the NAA2 was previously determined using 1D-TOCSY-MDEC and ROESY experiments and was assigned as 3R,5R,6S,7S.^24^ The diol bond between C5 and C6 was demonstrated to be cis configuration or has a free rotation making the bond susceptible to periodic acid (HIO_4_) cleavage.^25^ Further, we have previously reported that the sugar is d-xylose.^26^

### Production of Semisynthetic Analogues from the ASP7 Occidiofungin Variants (OCF-E and OCF-F)

The ASP7 variants afford an opportunity to efficiently produce semisynthetic analogues of occidiofungin by derivatizing the terminal carboxylic acid on the side chain of aspartic acid. Carbodiimide mediated condensation reaction is commonly used in peptide synthesis to create amide bonds between two amino acids.^27^ In this study, we prepared amides of ASP7 using a variety of primary amines, a secondary amine (dimethylamine), and ammonia. ASP7 variants (OCF-E and OCF-F) were incubated in the reaction mixture containing the amine of choice or ammonia in the presence of a carbodiimide. This afforded the amide analogues of occidiofungin. Seven analogues of occidiofungin were thus synthesized from this coupling reaction using ammonia, methylamine, dimethylamine, ethylamine, propylamine, but-3-yn-1amine, and dodecylamine. The products were isolated by analytical HPLC (Figure S8). Each reaction successfully produced the expected product as confirmed by HR-MS (Table 3). There were differences in yield and some amines led to the formation of side products (Figure S8). The condensation reaction of ASP7 variants with ammonia would result in the formation of ASN as position 7. The OCF-S2 product was shown to be identical by HR-MS to the naturally occurring variant OCF-B. However, the use of ammonia resulted in the formation of a significant amount of side products under the current reaction conditions and there was an unexpected product having an additional dehydration (loss of 18 Da). There was a significant amount of unreacted ASP7 variants following the reactions performed using propylamine, but-3-yn-1-amine, and dodecylamine. The desired products were isolated without any additional optimizations of the reaction condition used. This approach is agreeable to the generation of a large chemical library of occidiofungin analogues for screening purposes. The reactions can also be optimized in the future for scale-up preparation of analogues of interest. A schematic for the synthesis of semisynthetic peptides using the ASP7 variants is shown in the SI (Figure S9).

**Table 3:** Semi-Synthetic Occidiofungin Analogues Produced Using OCF-E and OCF-F Variants with Their Description, Theoretical Mass, and Observed Mass from HR-MS^a^

analogue variant and elemental composition	amine used	terminal group at position 7	amino acid at position 1	theoretical mass [M + H]^+^	observed mass [M + H]^+^	mass error (ppm)
OCF-S1 C_52_H_86_O_21_N_11_	ammonia NH_3_	amide (ASN variant)	ASN	1200.5994	not observed^b^	
OCF-S2 C_52_H_86_O_22_N_11_	ammonia NH_3_	amide (ASN variant)	BHN	1216.5943	1216.5972^c^	2.4
OCF-S3 C_53_H_87_O_21_N_11_	methylamine CH_3_NH_2_	*N*-methyl amide	ASN	1214.6151	1214.6183	2.6
OCF-S4 C_53_H_87_O_22_N_11_	methylamine CH_3_NH_2_	*N*-methylamide	BHN	1230.6100	1230.6122	1.8
OCF-S5 C_54_H_89_O_21_N_11_	dimethylamine (CH_3_)_2_NH	*N*-dimethylamide	ASN	1228.6307	1228.6330	1.9
OCF-S6 C_54_H_89_O_22_N_11_	dimethylamine (CH_3_)_2_NH	*N*-dimethylamide	BHN	1244.6256	1244.6270	1.1
OCF-S7 C_54_H_89_O_21_N_11_	ethylamine C_2_H_5_NH_2_	*N*-ethylamide	ASN	1228.6307	1228.6335	2.3
OCF-S8 C_54_H_89_O_22_N_11_	ethylamine C_2_H_5_NH_2_	*N*-ethylamide	BHN	1244.6256	1244.6275	1.5
OCF-S9 C_55_H_91_O_21_N_11_	propylamine C_3_H_9_N	*N*-propylamide	ASN	1242.6464	1242.6474	0.8
OCF-S10 C_55_H_91_O_22_N_11_	propylamine C_3_H_9_N	*N*-propylamide	BHN	1258.6413	1258.6421	0.6
OCF-S11 C_56_H_89_O_21_N_11_	but-3-yn-1-amine C_4_H_7_N	*N*-but-3-yn-1-amine	ASN	1252.6307	1252.6333	2.1
OCF-S12 C_56_H_89_O_22_N_11_	but-3-yn-1-amine C_4_H_7_N	*N*-but-3-yn-1-amine	BHN	1268.6256	1268.6276	1.6
OCF-S13 C_64_H_109_O_21_N_11_	dodecylamine CH_3_(CH_2_)_11_NH_2_	*N*-dodecylamide	ASN	1368.7872	1368.7893	1.5
OCF-S14 C_64_H_109_O_22_N_11_	dodecylamine CH_3_(CH_2_)_11_NH_2_	*N*-dodecylamide	BHN	1384.7821	1384.7850	2.1

a All observed masses obtained with HR-MS were within 5 ppm of the theoretical masses.

b A dehydrated product of the OCF-F variant was a single Dalton less than the expected mass of this OCF-S1analogue, The OCF-S1 analogue may be present but could be masked by the 1 Da+ isotope of the dehydrated product.

c Denotes unexpected mass of a side product (1199.5695) in the collected product.

HR-MS and NMR analyses of the semisynthetic methylamine analogues (OCF-S3 and OCF-S4) further confirmed the presence of the ASP at position 7 (Figures S10 and S11 and Table 4). A purified fraction of the semisynthetic methylamine analogue was used to prepare a sample for NMR analysis. The 2D TOCSY and NOESY spectra analyses showed a complete loss of the ASP7 spin systems, supporting the overall efficiency of the reaction (Figures S10 and S11). A new spin system with an H^N^ of 8.40 was present and overlapped with the assigned ASN7 amide spin system. The shift in the ASP7 spin system was due to the presence of the newly introduced *N*-methylamide to the side chain of aspartic acid (Figure S10). NOEs were observed between the H^α^ and H^β^ of *N*-methylamide ASP7 and H^N^ of SER8, and between H^α^ of GLY6 to the H^N^ of the *N*-methylamide ASP7 spin system. NOEs were observed between the H^N^ of *N*-methylamide ASP7 and the H^N^ of GLY6 and from the H^N^ of the attached methylamide group to the H^N^ of the *N*-methylamide ASP7 residue (Figure S11). In addition, NOEs were assigned between H^β^ of *N*-methylamide ASP7 residue and the γ-NH of the attached methylamide group. There are two distinct amide spin systems for the terminal methylamide, the γ-NH proton shifts of 7.87 and 7.82. The methyl protons had an average chemical shift value of 2.56. No significant alterations were observed for the spin systems of other amino acids.

**Table 4:** ^1^H NMR Chemical Shift Values for Methylamide-ASP7 Analogues of Occidiofungin (OCF-S3 and OCF-S4) in Dimethyl Sulfoxide (DMSO-d6) at 25°C

amino acid position	H^N^	H^α^	H^β^	other protons
ASN1	8.12	4.50	2.51, 2.37	γ-NH2:7.28, 6.87
	8.08	4.53	2.51, 2.34	γ-NH2:7.28, 6.87
BHN1	7.90	4.62	4.00	γ-NH2:7.32, 6.94 β-OH: 5.72
NAA2	7.54			C2-CH2:2.32, C3-CH: 4.14, C4-CH2:1.77, 1.37, C5-CH: 3.50, C5-OH: 4.18, C6-CH: 3.06, C6-OH:4.09, C7-CH: 3.75, C8-CH: 1.54, C9-C17-CH2:1.27, C18-CH3:0.85
	7.32			C2-CH2:2.32, C3-CH: 4.14, C4-CH2:1.77, 1.37, C5-CH: 3.50, C5-OH: 4.18, C6-CH: 3.06, C6-OH:4.09, C7-CH: 3.75, C8-CH: 1.54, C9-C17-CH2:1.27, C18-CH3:0.85
	7.28			C2-CH2:2.32, C3-CH: 4.14, C4-CH2:1.77, 1.37, C5-CH: 3.50, C5-OH: 4.18, C6-CH: 3.06, C6-OH:4.09, C7-CH: 3.75, C8-CH: 1.54, C9-C17-CH2:1.27, C18-CH3:0.85
				Xylose: C1-CH2:4.19, C2-CH: 2.99. C2-OH: 4.96, C3-CH:3:11, C3-OH: 4.96, C4-CH:3.30, C4-OH:4.98, C5-CH2:3.72, 3.06
SER3	8.16	4.13	3.43, 3.30	β-OH: 4.98
	8.09	4.19	3.46, 3.30	β-OH: 4.99
	7.98	4.62	3.80, 3.60	β-OH: 5.72
BHY4	7.95	4.14	5.06	β-OH: 5.76, OH – 9.33, C2&C6:CH – 7.15, C3&C5:CH – 6.67
DABA5	7.67	4.42	2.10, 1.90	γ-H: 2.90, NH2:7.75
GLY6	7.88	3.81, 3.68		
	7.70	3.91, 3.62		
ASN7	8.42	4.58	2.59, 2.40	γ-NH2:7.40, 6.94
	8.32	4.56	2.58, 2.40	γ-NH2:7.40, 6.94
GLN7	8.31	4.24	2.09, 1.89	ε-NH2:7.14, 7.04, C3-CH2:1.73
methylamide ASP7	8.40	4.58	2.58, 2.41	γ-NH: 7.87, 7.82, C6-CH3:2.56
SER8	7.90	4.62	3.82	β-OH: 4.83
	7.81	4.31	3.62	β-OH: 4.83

### Antifungal Activity of the ASP7 Occidiofungin Variants (OCF-E and OCF-F) and the Semisynthetic Analogues

Minimum inhibitory concentration (MIC) assays against three human fungal pathogen species, *C. albicans* ATCC3147, *C. glabrata* ATCC2001, and *C. auris* MYA-5001, were used to assess the activity of the ASP7 variants and the semisynthetic analogues synthesized using ammonia (OCF-S1and OCF-S2), methylamine (OCF-S3 and OCF-S4), dimethylamine (OCF-S5 and OCF-S6), ethylamine (OCF-S7 and OCF-S8), propylamine (OCF-S9 and OCF-S10), but-3-yn-1amine (OCF-S11 and OCF-S12), and dodecylamine (OCF-S13 and OCF-S14) (Table 5). *Candida* species are of serious clinical concern given the increase in antifungal resistance.^28,29^*C. glabrata* strains have a high prevalence of resistance to the azole class of antifungals.^30^ Furthermore, reports of multidrug resistant strains of *C. auris* have emerged in hospitals.^31,32^ Occidiofungin has been shown to be a potent antifungal against yeast^13^ and semisynthetic analogues of occidiofungin, with potentially better pharmacological properties, may help expand their use into other indications aside from the treatment of recurrent vulvovaginal candidiasis. Before these studies can be performed, we need to understand the effects of its bioactivity against yeast following the chemical modifications on the ASP7 residue. The ASP7 variants (OCF-E and OCF-F) have a low micromolar MIC against the three yeast strains tested. The activity of the ASP7 variants was 4- to 16-fold less than the inhibitory activity of the ASN7 variants (OCF-A and OCF-B). The OCF-S1 and OCF-S2 analogues synthesized using ammonia had identical HRMS masses as naturally occurring variants OCF-A and OCF-B. However, as mentioned previously, there were unexpected dehydration products in the collected fraction. These analogues, compared to the ASP7 variants, showed a 2-fold improvement in the MIC values against *C. albicans* and *C. glabrata* (Table 5). However, the activity was reduced compared to the inhibitory activity of OCF-A and OCF-B variants, presumably due to the presence of the unwanted side products. The methylamine analogues (OCF-S3 and OCF-S4) compared to the ASP7 variants had a 4-fold reduction in MICs against *C. albicans* and *C. glabrata* and a 2-fold decrease in MIC against *C. auris*. Similar improvements in the inhibitory activity against the yeast strains tested were observed with the dimethylamine (OCF-S5 and OCF-S6), propylamine (OCF-S7 and OCF-S8), and but-3-yn-1amine (OCF-S11 and OCF-S12) analogues. The but-3-yn-1-amine analogue still retained submicromolar activity and the analogue provides a functional alkyne group that can be used with azide–alkyne click chemistry.^33^ The alkyne analogue is potentially useful for the selective and high-throughput synthesis of additional semisynthetic analogues and in fluorescent microscopy experiments with azide fluorescent probes.^33^ The ethylamine (OCF-S7 and OCF-S8) products had the same level of activity as the starting substrates of the ASP7 variants. The range of semisynthetic analogues with antifungal activity shows that the ASP7 variants were amenable to the synthesis of a wide array of structurally distinct analogues with low micromolar activity (Table 5). The use of dodecylamine, to make the analogues OCF-S13 and OCF-S14, demonstrated this observation. The dodecylamine products demonstrated inhibitory activity against *C. glabrata* at low micromolar concentrations. There was a 4-fold increase in concentration compared to the inhibitory concentration of the ASP7 variants, while the strain of *C. albicans* was not inhibited at this concentration.

**Table 5:** Minimum Inhibitory Concentrations (μg/mL) of the Semi-Synthetic Occidiofungin Analogues against *C. albicans* ATCC3147, *C. glabrata* ATCC2001, and *C. auris* MYA-5001

minimum inhibitory concentration (μg/mL)			
occidiofungin variants and analogues	*C. albicans* ATCC3147	*C. glabrata* ATCC2001	*C. auris* MYA-5001
OCF-E and OCF-F^a^	2	2	1
ammonia (OCF-S1and OCF-S2)^b^	1	1	ND^c^
methylamine (OCF-S3 and OCF-S4)	0.5	0.5	0.5
dimethylamine (OCF-S5 and OCF-S6)	1	0.5	0.5
ethylamine (OCF-S7 and OCF-S8)	2	2	1
propylamine (OCF-S9 and OCF-S10)	0.5	0.5	1
but-3-yn-1-amine (OCF-S11 and OCF-S12)	0.5	0.5	ND
dodecylamine (OCF-S13 and OCF- S14)	>8	8	ND
OCF-A and OCF-B^a^	0.125	0.25	0.25

a OCF-B and OCF-F are the major naturally occurring variants produced by MS14.

b The isolated product for the ammonia reaction contained an additional side product having a dehydration, likely resulting in the observed loss in activity.

c ND = not determined.

The studies presented are a proof of concept for the use of ASP7 variants for the synthesis of novel analogues. Future studies will be performed to optimize the chemical reactions and to expand the structural library. The analogues can be screened for their use in other therapeutic applications.

## Experimental Section

### General Experimental Procedures

HR-MS was performed using an Orbitrap Fusion mass spectrometer (Thermo Fisher Scientific; Waltham, MA) or a Q Exactive Focus (Thermo Fisher Scientific; Waltham, MA). Each peak from the UHPLC purification of occidiofungin was analyzed by direct infusion on an Orbitrap Fusion mass spectrometer. Data was acquired in positive ion MS mode with a HESI probe set to a spray voltage of 3700 V. Semisynthetic analogues were confirmed by electrospray ionization mass spectrometry (ESI-MS) experiments using a Q Exactive Focus. Exactive Series 2.11/Xcalibur 4.2.47 software was used for data acquisition and processing. For NMR, a 3–4 mg sample of occidiofungin was dissolved in 600 μL of dimethyl sulfoxide (DMSO-d6; Cambridge Isotopes). The NMR data were collected on an Avance III HD-600 (Bruker; Billerica, MA), equipped with a CryoProbe, operating at a proton frequency of 600 MHz. The spectral sweep width for the TOCSY and NOESY is 18 ppm in both dimensions and centered at the water peak at about 3.3 ppm. The TOCSY and NOESY 2D data sets were collected with 2048 complex points in the acquisition dimension and 256 complex points for the indirect dimensions. Data was processed with nmrPipe and analyzed with the interactive computer program NMRView.^34,35^ The Marfey’s derivatization was performed on the sample hydrolysate by previously reported methods.^21^ The resulting derivatives from the sample and the derivatives of standard amino acids were analyzed by HPLC.

### Extraction and Isolation

Isolation of occidiofungin was performed as previously reported with some modifications.^8^ The producing strain, *B. contaminans* MS14, was grown in modified minimal M9 media with the glucose carbon source substituted for asparagine (1 g/L). The final pH of the medium was around 6.9. A 10% inoculum with an O.D._600_ between 0.6 and 0.8 was made before incubating the culture at 28 °C for 3 to 4 days. Cell free extracts of the culture were passed through a 0.2 μm filter before preparative HPLC purification. A 5–7 mL sample containing occidiofungin (∼30 mg) in 35:65 (acetonitrile/water), was loaded onto a SinoChrom ODS-BP 5 μm 20 × 150 mm^2^ column using a CHEETAH Preparative HPLC system (Bonna-Agela, QBH P100). The mobile phase consisted of acetonitrile (A) and double-distilled water (B), both with 0.1% formic acid. The gradient was set up as follows: held at 5% A for 5 min, from 5% to 50% A over 11 min, held at 50% A for 10 min, from 50% to 90% A over 10 min, and re-equilibrated by running at 5% A for 5 min. The occidiofungin fraction eluted out during the 50% A isocratic hold. The preparative HPLC fractions were checked by mass spectrometry analyses before the samples were freeze-dried on a benchtop freeze-dryer (Labconco; Kansas City MO). A stock of occidiofungin at 1 mg/mL in 35:65 (acetonitrile:water) was prepared for additional chromatography and mass spectrometry analyses.

### Occidiofungin Variants

#### Occidiofungin Variant OCF-A (1)

Amorphous white powder; UV (water) λ_max_ 220; ^1^H and ^13^C NMR data (DMSO-*d*_6_), Table 2; HR-MS *m*/*z* 1200.6027 [M + H]^+^ (calcd for C_52_H_86_O_21_N_11_) see Table 1.

#### Occidiofungin Variant OCF-B (2)

Amorphous white powder; UV (water) λ_max_ 220; ^1^H and ^13^C NMR data (DMSO-*d*_6_), Table 2; HR-MS *m*/*z* 1216.5974 [M + H]^+^ (calcd for C_52_H_86_O_22_N_11_) see Table 1.

#### Occidiofungin Variant OCF-E (3)

Amorphous white powder; UV (water) λ_max_ 220; ^1^H and ^13^C NMR data (DMSO-*d*_6_), Table 2; HR-MS *m*/*z* 1201.5874 [M + H]^+^ (calcd for C_52_H_85_O_22_N_10_) see Table 1.

#### Occidiofungin Variant OCF-F (4)

Amorphous white powder; UV (water) λ_max_ 220; ^1^H and ^13^C NMR data (DMSO-*d*_6_), Table 2; HR-MS *m*/*z* 1217.5824 [M + H]^+^ (calcd for C_52_H_85_O_23_N_10_) see Table 1.

#### Occidiofungin Variant OCF-H (5)

Amorphous white powder; UV (water) λ_max_ 220; ^1^H and ^13^C NMR data (DMSO-*d*_6_), Table 2; HR-MS *m*/*z* 1230.6134 [M + H]^+^ (calcd for C_53_H_88_O_22_N_11_) see Table 1.

#### Occidiofungin Variant OCF-J (6)

Amorphous white powder; UV (water) λ_max_ 220; HR-MS *m*/*z* 1231.5983 [M + H]^+^ (calcd for C_53_H_87_O_23_N_10_) see Table 1.

A 10 μL sample was loaded onto a Phenomenex BioZen 1.6 μM, Peptide PS-C18, 150 × 2.1 mm^2^ column on an ultrahigh-performance liquid chromatography (UHPLC; Shimadzu LC-2040C). The mobile phase consisted of acetonitrile (A) and double-distilled water (B), both with 0.1% formic acid. The gradient was set up as follows: held at 25% A for 2 min, from 25% to 40% A over 14 min, from 40% to 95% A over 30 s, held at 95% A for 3 min, from 95% to 25% A over 24 s, and held again at 25% A for 5 min to equilibrate the column for the next run. The substance separated into three major peaks between 10 and 13.6 min (Figure S3).

### Carbodiimide-Mediated Condensation

The carbodiimide mediated condensation reaction was performed with 1-ethyl-3-(3-(dimethylamino)propyl) carbodiimide hydrochloride (EDC). The ASP7 occidiofungin variants were incubated with a 10-fold excess molar ratio of the desired amine, EDC, and 1-hydroxybenzotriazole (HOBt) in *N*–*N*-dimethylformamide (DMF) or dimethyl sulfoxide (DMSO) at room temperature for at least 16 h. *N*–*N*-Dimethylformamide was used for the condensation reaction with ammonia and dimethyl sulfoxide was used for all other reactions. After the incubation period, the reaction mixture was brought up to 1 mL by dilution with a mixture of 35:65 (acetonitrile/water with 0.1% TFA). The resulting solution was loaded onto a Supercell ODS2 5 μm 4.6 × 250 mm^2^ column using a DuoFlow HPLC with a flow rate of 1 mL/min (Bio-Rad Laboratories, Hercules, California). The mobile phase consisted of acetonitrile (A) and double-distilled water (B), both with 0.1% trifluoroacetic acid. The gradient was set up as follows: from 10% A to 80% over 30 min and re-equilibrated by running at 10% A for 5 min. The amount of each analogue was quantified by comparing the fraction to the peak area of a 50 μg occidiofungin standard.

### Occidiofungin Semisynthetic Analogues OCF-S2–OCF-S14

#### Occidiofungin Analogue OCF-S2 (8)

Amorphous white powder; UV (water) λ_max_ 220; HR-MS *m*/*z* 1217.5784 [M + H]^+^ (calcd for C_52_H_86_O_22_N_11_) see Table 3.

#### Occidiofungin Analogue OCF-S3 (9)

Amorphous white powder; UV (water) λ_max_ 220; ^1^H and ^13^C NMR data (DMSO-*d*_6_), Table 4; HR-MS *m*/*z* 1214.6183 [M + H]^+^ (calcd for C_53_H_87_O_21_N_11_) see Table 3.

#### Occidiofungin Analogue OCF-S4 (10)

Amorphous white powder; UV (water) λ_max_ 220; ^1^H and ^13^C NMR data (DMSO-*d*_6_), Table 4; HR-MS *m*/*z* 1230.6122 [M + H]^+^ (calcd for C_53_H_87_O_22_N_11_) see Table 3.

#### Occidiofungin Analogue OCF-S5 (11)

Amorphous white powder; UV (water) λ_max_ 220; HR-MS *m*/*z* 1228.6330 [M + H]^+^ (calcd for C_54_H_89_O_21_N_11_) see Table 3.

#### Occidiofungin Analogue OCF-S6 (12)

Amorphous white powder; UV (water) λ_max_ 220; HR-MS *m*/*z* 1244.6270 [M + H]^+^ (calcd for C_54_H_89_O_22_N_11_) see Table 3.

#### Occidiofungin Analogue OCF-S7 (13)

Amorphous white powder; UV (water) λ_max_ 220; HR-MS *m*/*z* 1228.6335 [M + H]^+^ (calcd for C_54_H_89_O_21_N_11_) see Table 3.

#### Occidiofungin Analogue OCF-S8 (14)

Amorphous white powder; UV (water) λ_max_ 220; HR-MS *m*/*z* 1244.6275 [M + H]^+^ (calcd for C_54_H_89_O_22_N_11_) see Table 3.

#### Occidiofungin Analogue OCF-S9 (15)

Amorphous white powder; UV (water) λ_max_ 220; HR-MS *m*/*z* 1242.6474 [M + H]^+^ (calcd for C_55_H_91_O_21_N_11_) see Table 3.

#### Occidiofungin Analogue OCF-S10 (16)

Amorphous white powder; UV (water) λ_max_ 220; HR-MS *m*/*z* 1258.6421 [M + H]^+^ (calcd for C_55_H_91_O_22_N_11_) see Table 3.

#### Occidiofungin Analogue OCF-S11 (17)

Amorphous white powder; UV (water) λ_max_ 220; HR-MS *m*/*z* 1252.6333 [M + H]^+^ (calcd for C_56_H_89_O_21_N_11_) see Table 3.

#### Occidiofungin Analogue OCF-S12 (18)

Amorphous white powder; UV (water) λ_max_ 220; HR-MS *m*/*z* 1268.6276 [M + H]^+^ (calcd for C_56_H_89_O_22_N_11_) see Table 3.

#### Occidiofungin Analogue OCF-S13 (19)

Amorphous white powder; UV (water) λ_max_ 220; HR-MS *m*/*z* 1368.7893 [M + H]^+^ (calcd for C_64_H_109_O_21_N_11_) see Table 3.

#### Occidiofungin Analogue OCF-S14 (20)

Amorphous white powder; UV (water) λ_max_ 220; HR-MS *m*/*z* 1384.7850 [M + H]^+^ (calcd for C_64_H_109_O_22_N_11_) see Table 3.

### Biological Assays

The antifungal activity of the ASP7 variants and the semisynthetic analogues was tested. A modified CLSI M27-A3 method using yeast extract peptone dextrose (YPD) growth media was used for minimum inhibitory concentration (MIC) assays. The plates were incubated at 35 °C for 24 h, and the MIC values were determined by the absence of visible growth in individual wells on the plate at 24 h. Three clinically relevant species of *Candida* were tested, *C. albicans* ATCC3147, *C. glabrata* ATCC2001, and *C. auris* MYA-500.

## Supporting Information Available

Schemes, chromatography of variants and semisynthetic analogues, and 2D NMR spectra for occidiofungin and *N*-methylamide-aspartic acid analogue (PDF)
