# Supplementary material for: Previously Uncharacterized Variants, OCF-E–OCF-J, of the Antifungal Occidiofungin Produced by Burkholderia contaminans MS14
Source: J Nat Prod. 2024 Jan 26;87(2):186–94. doi: 10.1021/acs.jnatprod.3c00777 (PMC10897925; doi:10.1021/acs.jnatprod.3c00777)
Supplement: Supplementary file 1 — np3c00777_si_001.pdf [file np3c00777_si_001.pdf]

Previously Uncharacterized Variants, OCF-E – OCF-J, of the Antifungal Occidiofungin Produced by *Burkholderia contaminans* MS14

Nopakorn Hansanant[1], Kevin Cao[2], Abraham Tenorio[2], Thushinari Joseph[1], Min Ju[1], Noah McNally[1], Evangel Kummari[1], McKinley Williams[1], Andrew Cothrell[1], Andrew R. Buhrow[1], Ronald Shin[3], Ravi Orugunty[2], James L. Smith[1,2*]

[1]*Department of Biology, Texas A&M University, College Station, TX, United States*
[2]*Sano Chemicals Inc., Bryan, TX, United States*
[3]*Central Alabama High-Field NMR Facility, Structural Biology Shared Facility, Cancer Center, University of Alabama at Birmingham, Birmingham, Alabama, USA*

*Corresponding Authors

E-mail: jsmith@bio.tamu.edu (L. S.).

E-mail: rorugunty@sanochemicals.com (R. O.).

Table S1. ^{1}H NMR chemical shift values for the Asp7 variants (OCF-E and OCF-F) in dimethyl sulfoxide (DMSO-d6) at 25°C.
Figure S1. Proposed biosynthetic pathway of occidiofungin.
Figure S2. Preparative HPLC chromatogram of a purified occidiofungin fraction.
Figure S3. Isolation of occidiofungin variants.
Figure S4. TOCSY NMR Spectra of occidiofungin.
Figure S5. NOESY data showing NOE interactions.
Figure S6. TOCSY NMR Spectra of the aspartic acid occidiofungin variants E and F
Figure S7. Marfey's derivatization analysis of occidiofungin
Figure S8. HPLC chromatograms of each semi-synthetic analogue.
Figure S9. Reaction schematic for the synthesis of novel semi-synthetic analogues of occidiofungin.
Figure S10. Overlays of the N-methylamide-ASP7 semi-synthetic variants (OCF-S3 and OCF-S4) 2D NMR data.
Figure S11. Amide to Amide NOESY NMR spectra.
Figure S12. 2D TOCSY Spectra of occidiofungin preparative fraction
Figure S13. 2D NOESY Spectra of occidiofungin preparative fraction
Figure S14. 2D TOCSY Spectra of N-methylamine-aspartic acid analogue (OCF-S3 and OCF-S4)
Figure S15. 2D NOESY Spectra of N-methylamine-aspartic acid analogue (OCF-S3 and OCF-S4)
Figure S16. 2D TOCSY Spectra of Asp7 variants (OCF-E and OCF-F)
Figure S17. 2D NOESY Spectra of Asp7 variants (OCF-E and OCF-F)

Table S1. ^{1}H NMR chemical shift values for the Asp7 variants (OCF-E and OCF-F) in dimethyl sulfoxide (DMSO-d6) at 25°C.

**Amino Acid Position**	**H^N**	**H^α**	**H^β**	**Other protons**
ASN1	*8.09*	*4.52*	*2.52, 2.35*	γ-NH2: 7.26, 6.86
BHN1	*7.90*	*4.63*	*4.02*	γ-NH2: 7.30, 6.93 β-OH: 5.70
NAA2	*7.53*			C2-CH2: 2.32, 2.40 C3-CH: 4.15, C4-CH2: 1.76, 1.35, C5-CH: 3.51, C5-OH: 4.17, C6-CH: 3.05, C6-OH:4.08, C7-CH: 3.75, C8-CH: 1.36, C9-C17-CH2: 1.31, C18-CH3: 0.86
NAA2	*7.28*			C2-CH2: 2.32, 2.40 C3-CH: 4.15, C4-CH2: 1.76, 1.35, C5-CH: 3.51, C5-OH: 4.17, C6-CH: 3.05, C6-OH:4.08, C7-CH: 3.75, C8-CH: 1.36, C9-C17-CH2: 1.31, C18-CH3: 0.86
				Xylose: C1-CH2:4.18, C2-CH: 2.99, C2-OH: 4.94 C3-CH:3:10, C3-OH: 4.94, C4-CH:3.30, C4-OH:4.93, C5-CH2: 3.72,3.06
SER3*	*8.07*	*4.18*	*3.42*	*β-OH: 4.97*
SER3	*8.15*	*4.14*	*3.43, 3.30*	*β-OH: 4.94*
BHY4	*7.95*	*4.14*	*5.09*	*β-OH: 5.73, OH: 9.33, C2&C6-CH:7.15, C3&C5-CH: 6.68*
DABA5	*7.65*	*4.42*	*2.11, 1.89*	*γ-H: 2.92, NH2: 7.75*
GLY6*	*7.98*	*3.81, 3.64*		
GLY6	*7.85*	*3.83, 3.63*		
ASP7*	*8.41*	*4.57*	*2.76, 2.50*	
ASP7	*8.47*	*4.58*	*2.76, 2.50*	
SER8*	*7.80*	*4.23*	*3.58*	*β-OH: 4.83*
SER8	*7.85*	*4.34*	*3.60*	*β-OH: 4.82*

*** Demarcates the spin systems for the Asp7 and Asn1 variant (OCF-E).**

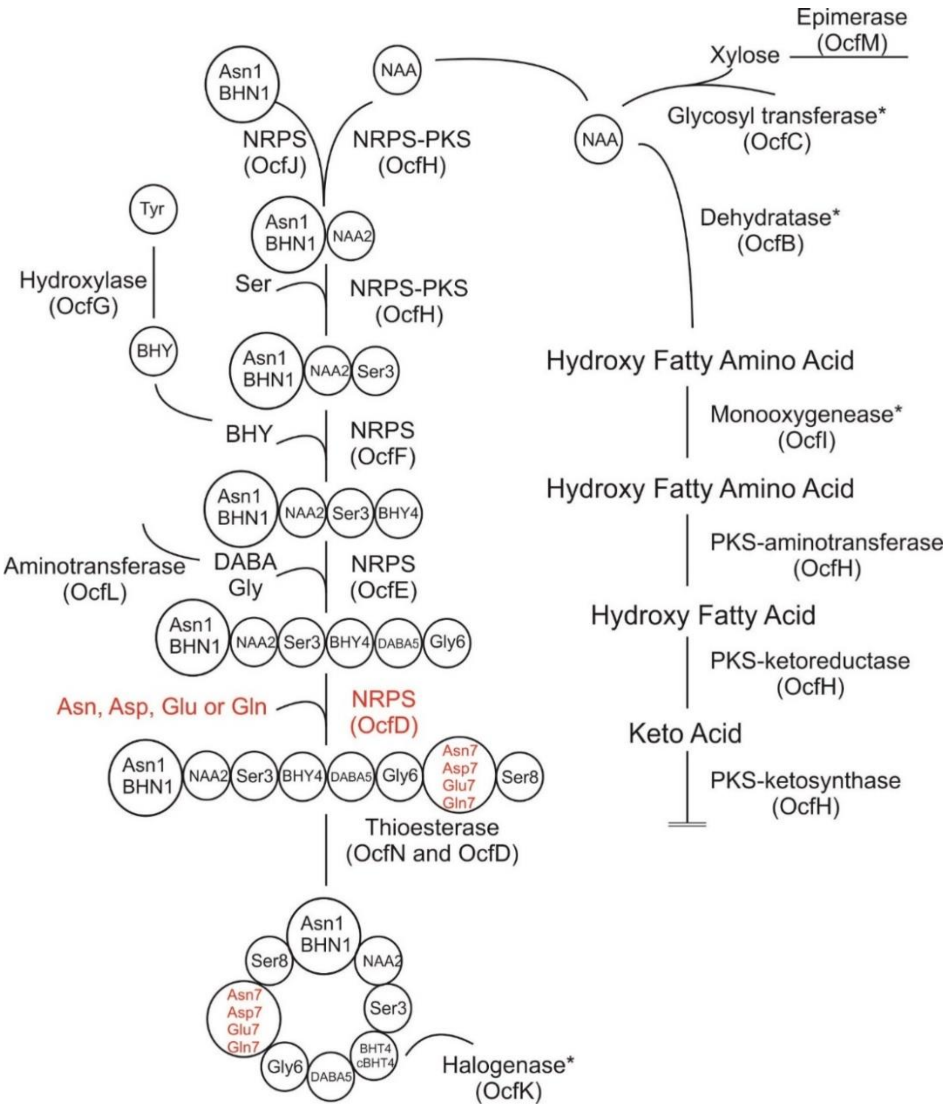

Figure S1. Proposed biosynthetic pathway of occidiofungin. A polyketide synthesis pathway is responsible for the synthesis of Novel amino acid 2 (NAA2). NAA2 is subsequently attached to the peptide in the non-ribosomal synthesis pathway. OcfD (highlighted in red) is responsible for the addition of the seventh residue and may have promiscuity in selectivity. Aside from previously reported ASN, ASP, GLU and GLN are the additional amino acids that have been demonstrated to be introduced at the seventh position. Steps demarcated with an asterisk show potential alternative synthesis routes.

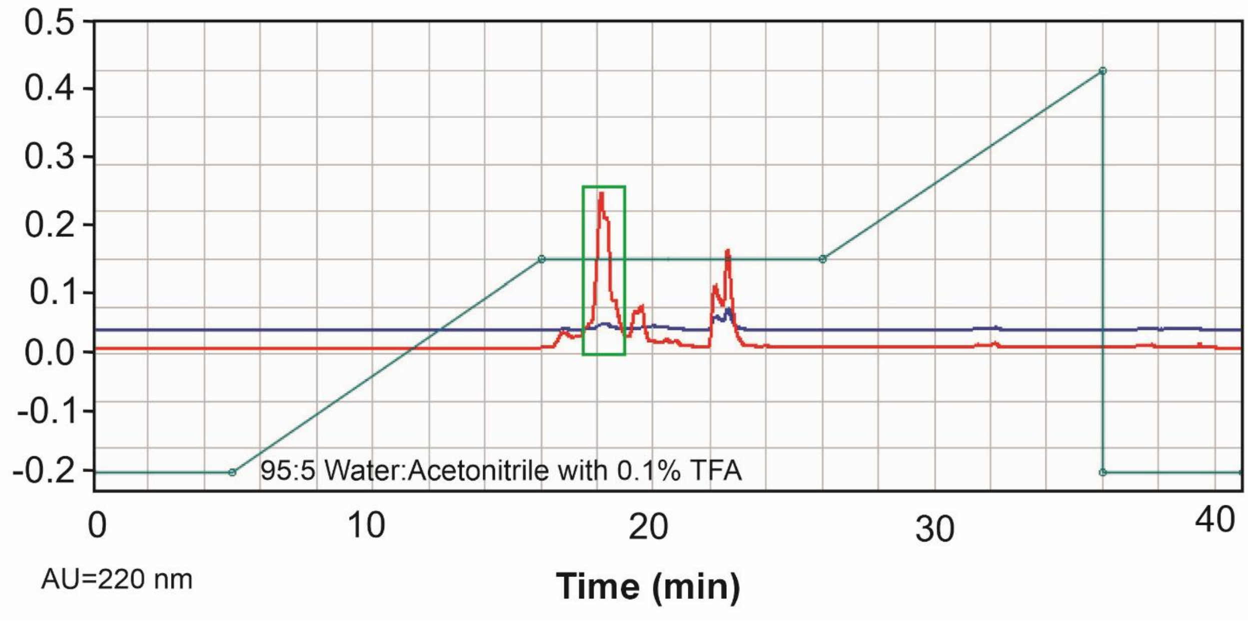

Figure S2. Preparative HPLC chromatogram of a purified occidiofungin fraction. Occidiofungin was isolated using a 20mm X 150mm C18 SinoChrom ODS-BP 5 µm column. The green box highlights the preparative HPLC fraction of occidiofungin. The red line is absorbance at 220 nm, while the purple line is absorbance at 280 nm. Green line represents the percentage of acetonitrile.

A

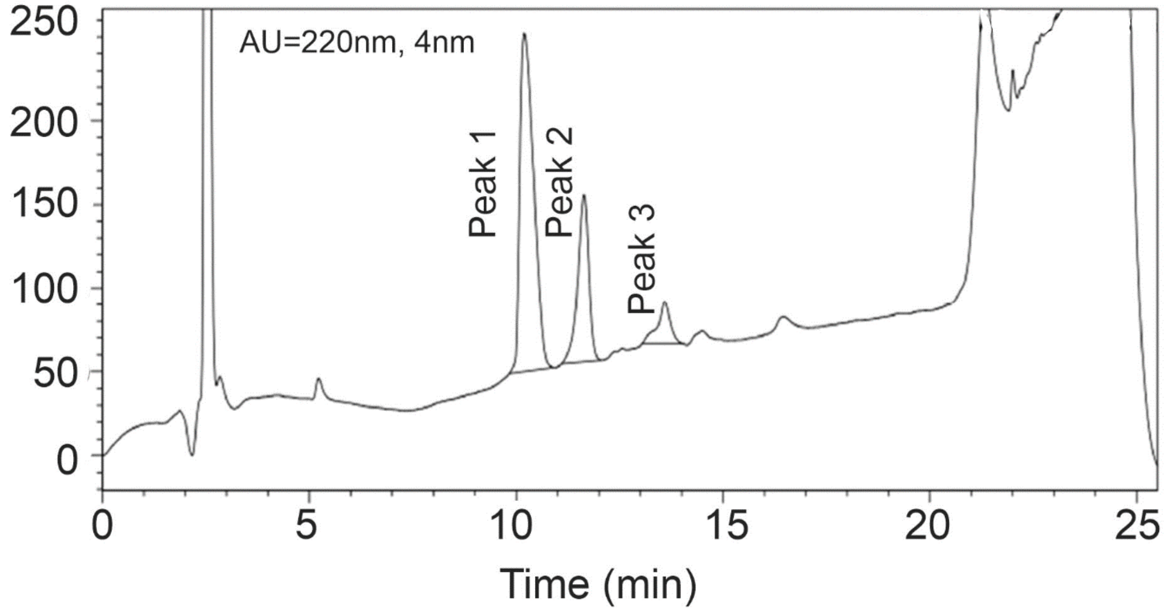

B

	Observed Occidiofungin Variants	Average Retention Time (min)	Average Peak Area (AU)	Average Relative Percent Composition (%)
Peak 1	OCF-A, OCF-B	10.20	4776722	66.4
Peak 2	OCF-E, OCF-F	11.65	1873212	26.0
Peak 3	OCF-H	13.59	544572	7.6

Figure S3. Isolation of occidiofungin variants. **A)** Representative chromatogram of the ultra-performance liquid chromatography (UPLC) separation of occidiofungin variants. Preparative fraction of occidiofungin was chromatographed on a Phenomenex bioZen 1.6μM, Peptide PS-C18, 50 × 2.1 mm column. Peak 1 eluted at 10.201 minutes, peak 2 eluted at 11.643 minutes and peak 3 eluted at 13.592 minutes. **B)** The table showed the average peak area and the percent relative composition of each peak from 3 separate runs. The occidiofungin variants in each peak was also noted in the table.

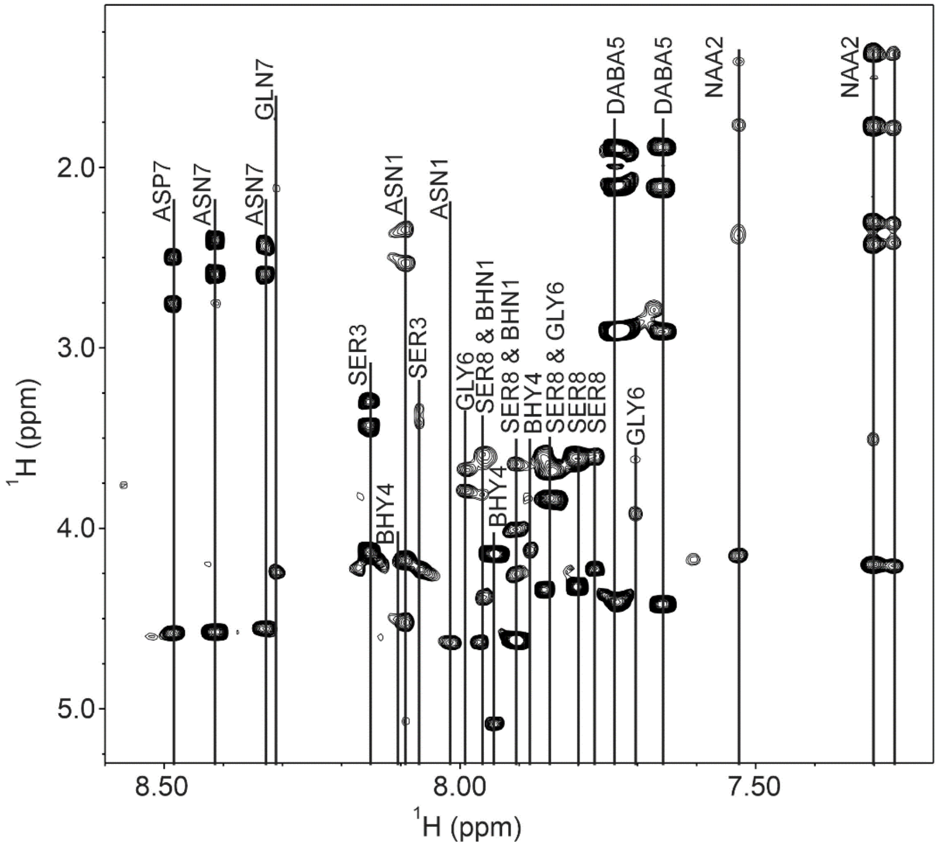

Figure S4. TOCSY NMR Spectra of occidiofungin. Expansion of the TOCSY 2D NMR spectra showing the amide to alpha, and amide to side chain spin systems for each assigned residue in occidiofungin fraction shown in Figure S2. The new spin systems for the presence of aspartic acid and glutamine at position 7 are shown.

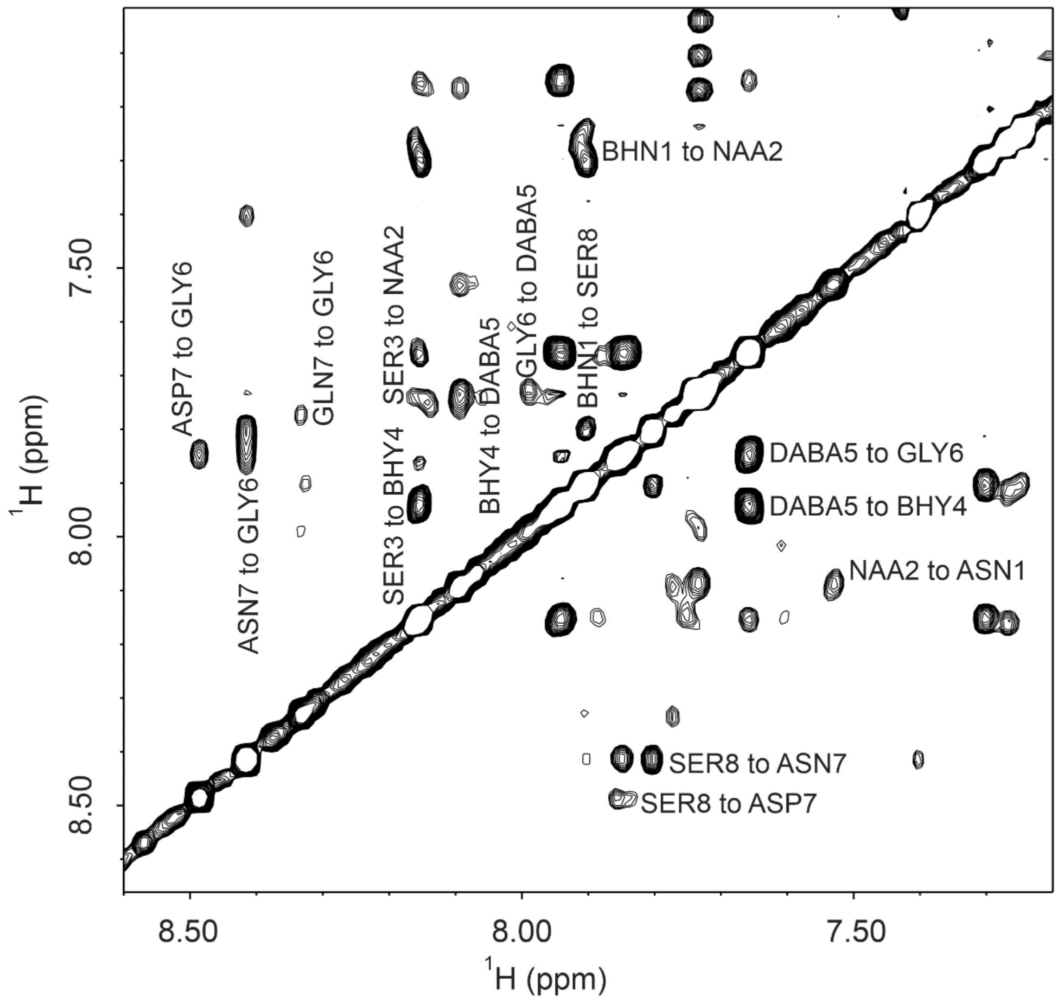

Figure S5. NOESY data showing NOEs from H^N of GLY6 to the H^N of the ASP7, ASN7, and GLN7 spin systems. NOEs from HN of SER8 to HN of ASP7 and ASN7 are shown. A complete sequential walk from each amino acid in the cyclic peptide is observed in the amide-to-amide interactions.

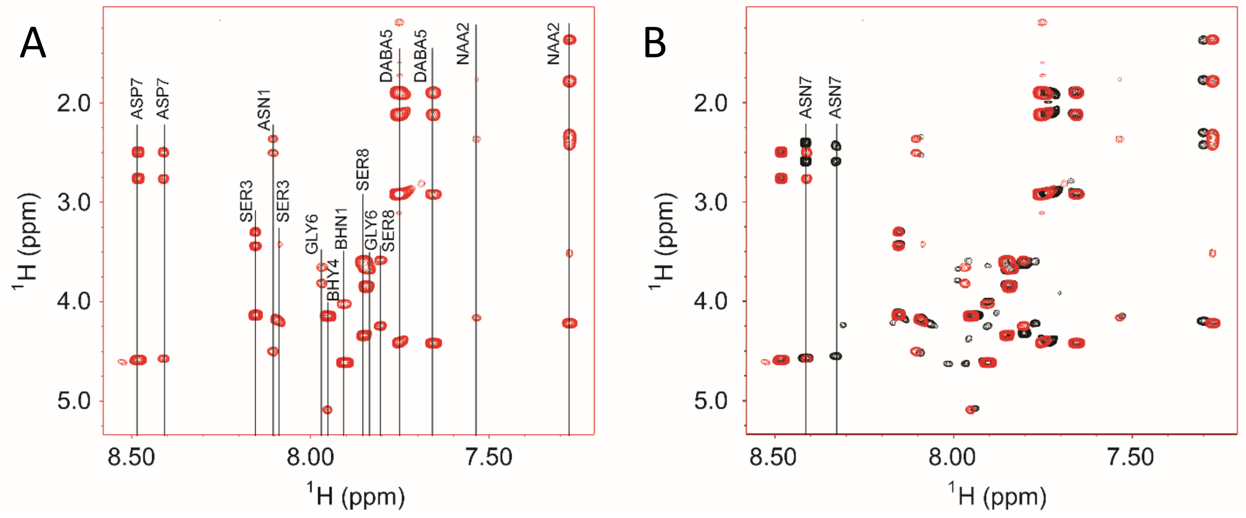

Figure S6. TOCSY NMR Spectra of the aspartic acid occidiofungin variants E and F. **A)** Expansion of the TOCSY 2D NMR spectra showing the amide to alpha, and amide to side chain spin systems for each assigned residue in the aspartic acid fraction. **B)** The fingerprint region of the isolated aspartic acid fraction is overlaid with the fingerprint region of the fraction shown in Figure S2. The Asp7 analogues are visible while the Asn7 analogues are absent, supporting the residue assignments.

A

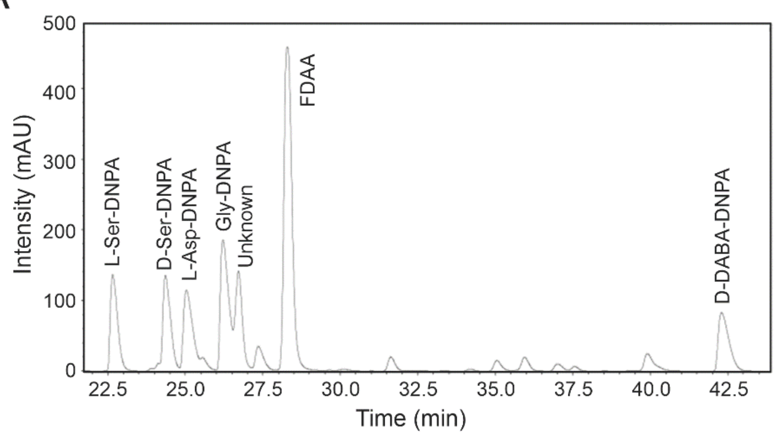

B

Amino Acid Standards

Amino Acid Derivative	RT (min)
L-Ser-DNPA	22.6
D-Ser-DNPA	24.3
L-Asp-DNPA	25.0
Gly-DNPA	26.3
D-Asp-DNPA	27.1
FDAA	28.5
L-DABA-DNPA	39.7
D-DABA-DNPA	41.6

OCF Reaction Mixture

Amino Acid Derivative	RT (min)
L-Ser-DNPA	22.7
D-Ser-DNPA	24.4
L-Asp-DNPA	25.0
Gly-DNPA	26.2
Unknown	26.7
FDAA	28.3
D-DABA-DNPA	42.3

Figure S7. Marfey's derivatization analysis of occidiofungin. **A.** The Marfey's derivatization of the acid hydrolysis of the natural product was run using an optimized HPLC method (UV 340 nm) with a C18 Gemini-MX 3 micron 150 × 2 mm ID column. Acetonitrile and 10 mM ammonium formate, pH 4.0 was used. A gradient of 5-60% acetonitrile over a 45-minute run time at 0.2 mL/min was used. **B.** The retention time for both the amino acid standards and the hydrolysis mixture of OCF. The retention time for each amino acid standard was compared to the retention times of the hydrolysis of the OCF product. FDAA (1-fluoro-2-4-dinitrophenyl-5-L-alanine amide) was used as the Marfey's reagent. An unknown peak was observed at 26.7 min and could possibly be a degradation product of one of the other non-conventional amino acids.

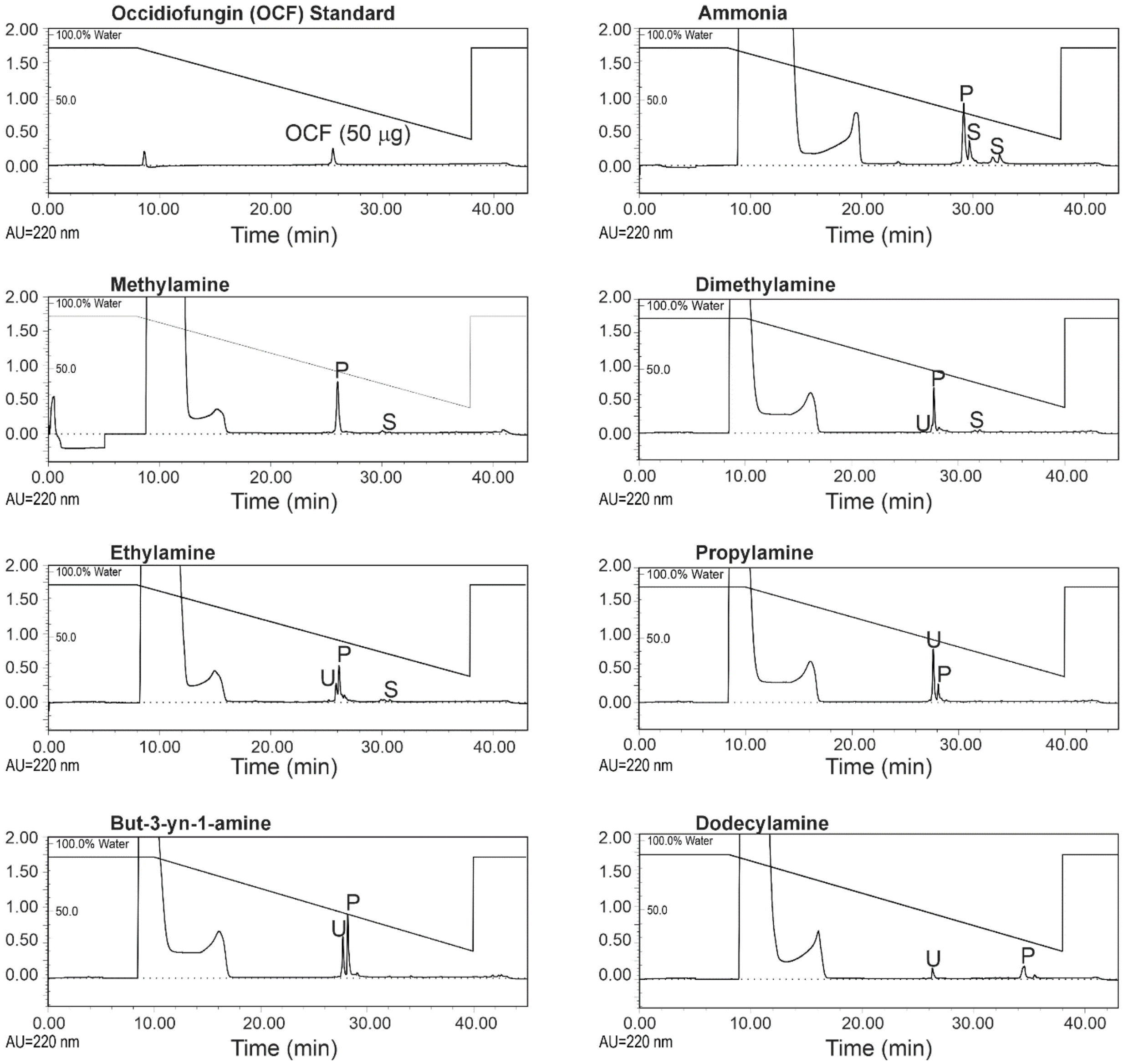

Figure S8. HPLC chromatograms of each semi-synthetic analogue. A standard of 50 µg of occidiofungin on column was used for determining the amount of product (P) for subsequent bioassays. The unreacted material (U) and side product material (S) are noted. There was a side product (S) that eluted with the product (P) for the ammonia reactions and was observed in the HR-MS analysis. The product for the ammonia reaction contained a side product having an additional dehydration.

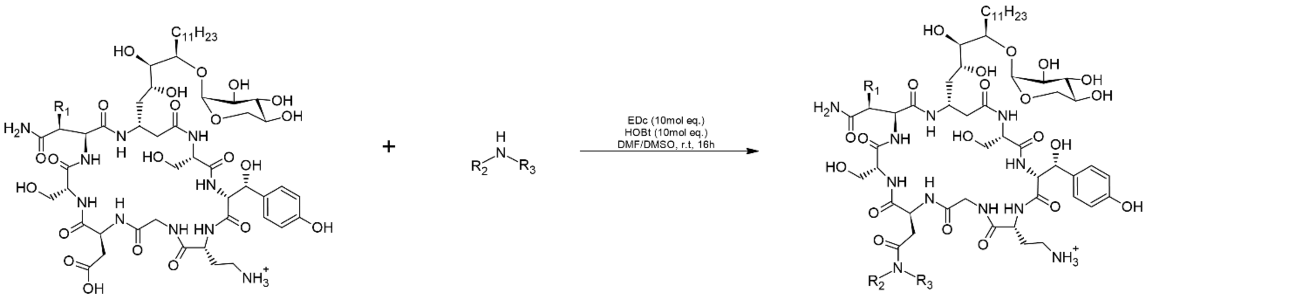

R_1=H, OH

R_2=H	R_3=H
R_2=CH_3	R_3=H
R_2=CH_3	R_3=CH_3
R_2=CH_2CH_3	R_3=H
R_2=$(CH_2)_2CH_3$	R_3=H
R_2=$(CH_2)_2CCH$	R_3=H
R_2=$(CH_2)_{11}CH_3$	R_3=H

R_1=H, OH

R_2=H	R_3=H	OCF-S2
R_2=CH_3	R_3=H	OCF-S3, OCF-S4
R_2=CH_3	R_3=CH_3	OCF-S5, OCF-S6
R_2=CH_2CH_3	R_3=H	OCF-S7, OCF-S8
R_2=$(CH_2)_2CH_3$	R_3=H	OCF-S9, OCF-S10
R_2=$(CH_2)_2CCH$	R_3=H	OCF-S11, OCF-S12
R_2=$(CH_2)_{11}CH_3$	R_3=H	OCF-S13, OCF-S14

Figure S9. Reaction schematic for the synthesis of novel semi-synthetic analogues of occidiofungin. The reaction depicts a carbodiimide-mediated condensation reaction of the ASP7 occidiofungin variants (OCF-E and OCF-F) with a primary or secondary amine or ammonia. A 10 molar equivalent of 1-ethyl-3-(3-dimethylaminopropyl) carbodiimide hydrochloride) (EDC), 1-hydroxybenzo-triazole (HOBt) and the amine or ammonia was added to the ASP7 variants. The reaction was carried out in anhydrous N,N-dimethylformamide, or dimethyl sulfoxide . The reaction was incubated at room temperature for at least 16 hours before separation via HPLC.

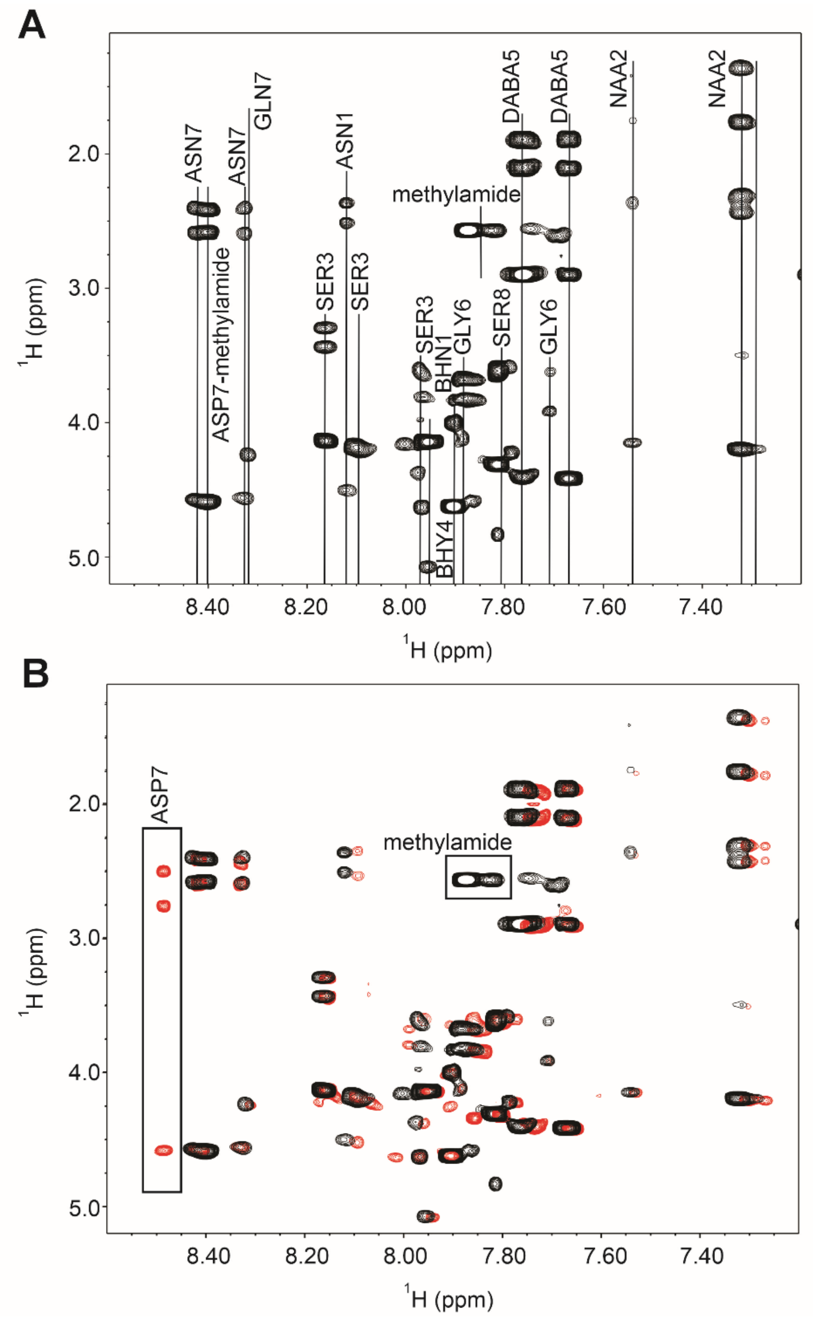

Figure S10. NMR data showing **A)** The TOCSY fingerprint region of the N-methylamide-ASP7 semi-synthetic variants (OCF-S3 and OCF-S4). The spin system for ASP7 is no longer present, and a new spin system assigned as N-methylamide was present, indicating that OCF-E and OCF-F were fully converted during the EDC condensation reaction. Spin systems for the other amino acids present in occidiofungin are also labeled. **B)** Overlay of the TOCSY fingerprint region of the preparative HPLC fraction (in red) of occidiofungin with the N-methylamide-ASP7 semi-synthetic product (in black). The ASP7 spin system is absent in the product of the methylamine EDC coupling reaction. The addition of the methylamine does cause some small amide shifts for the other amino acids.

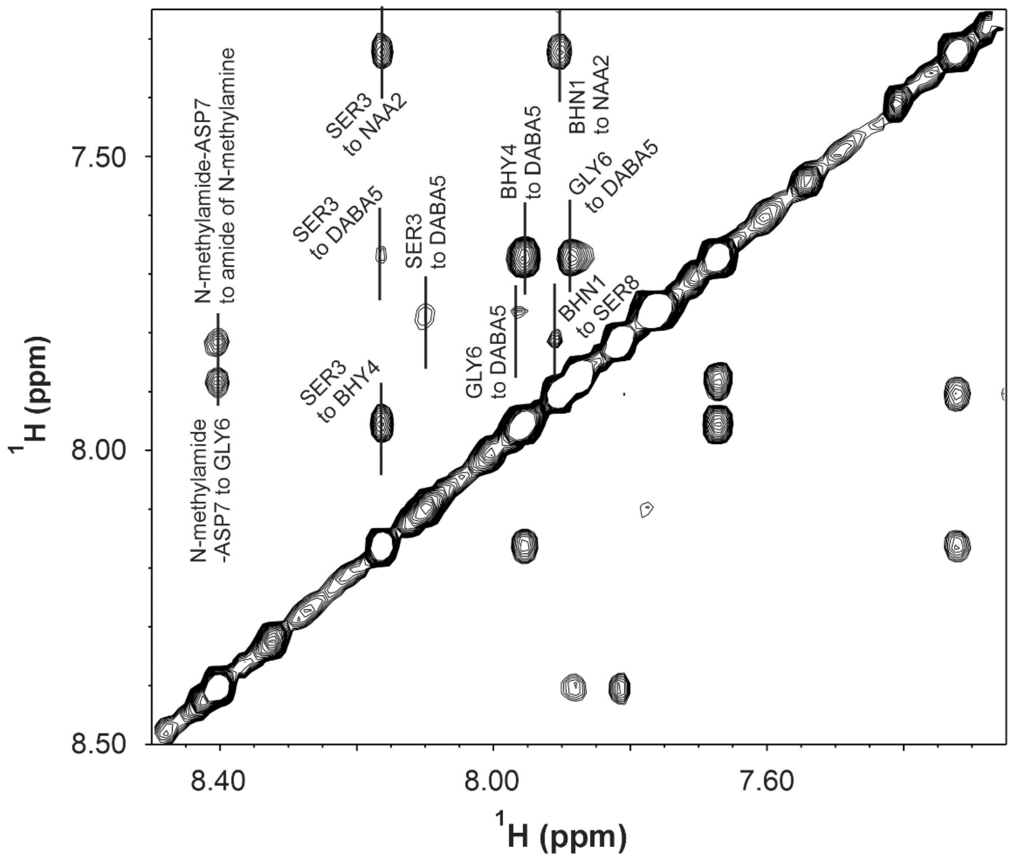

Figure S11. Amide to Amide NOESY NMR spectra. The NOESY data shows the NOEs between H^N of the ASP7 residue to the γ-NH of the N-methylamide. Amide to amide interactions are also observed between GLY6 and N-methylamide-ASP7 analogue. An almost complete sequential walk can be made from the amide to amide proton interactions. NOEs are seen indicating interaction of H^α of Gly6 to the H^N of the N-methylamide7 spin system and H^α and H^β of the N-methylamide7 spin system and H^N of SER8. These additional NOEs in the NOESY spectra enable a complete sequential walk for all the amino acids in the peptide.

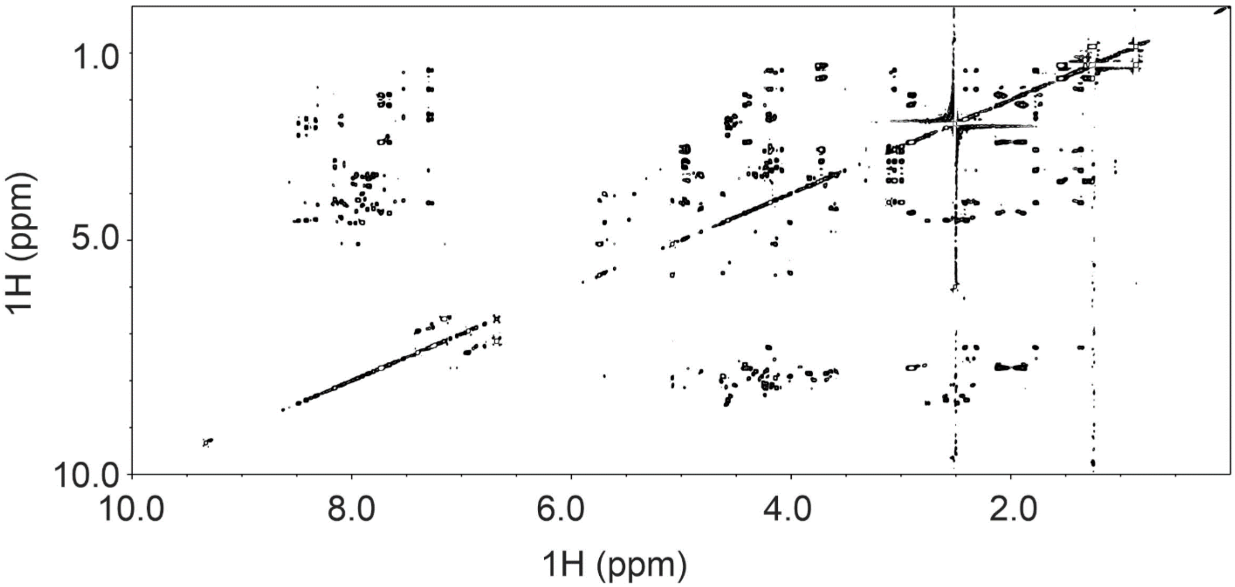

Figure S12. 2D TOCSY Spectra of occidiofungin preparative fraction

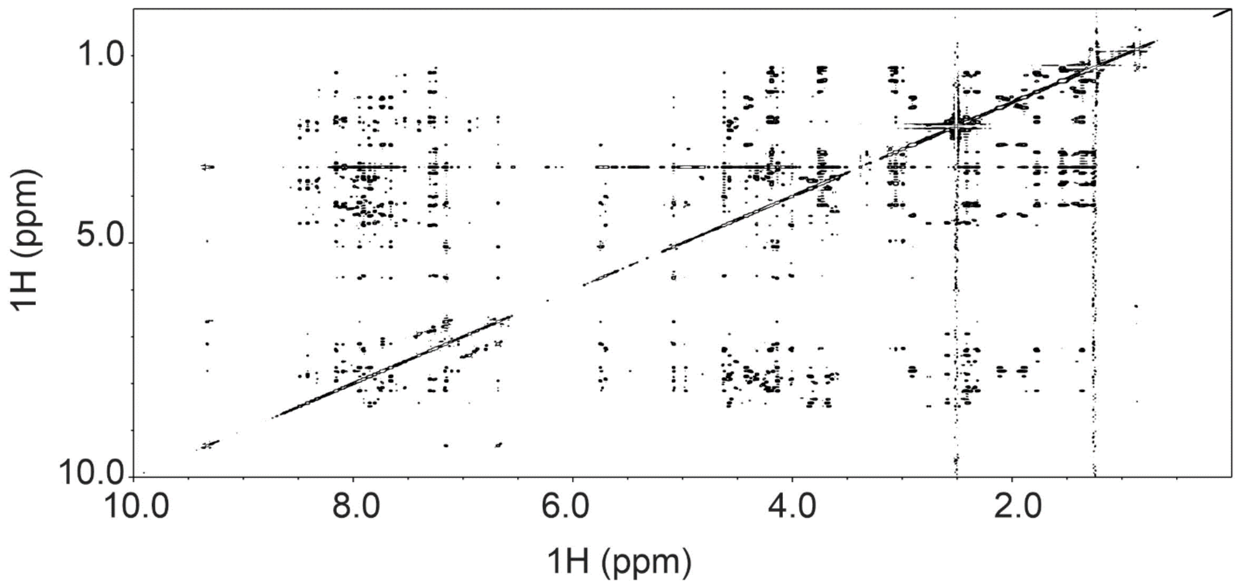

Figure S13. 2D NOESY Spectra of occidiofungin preparative fraction

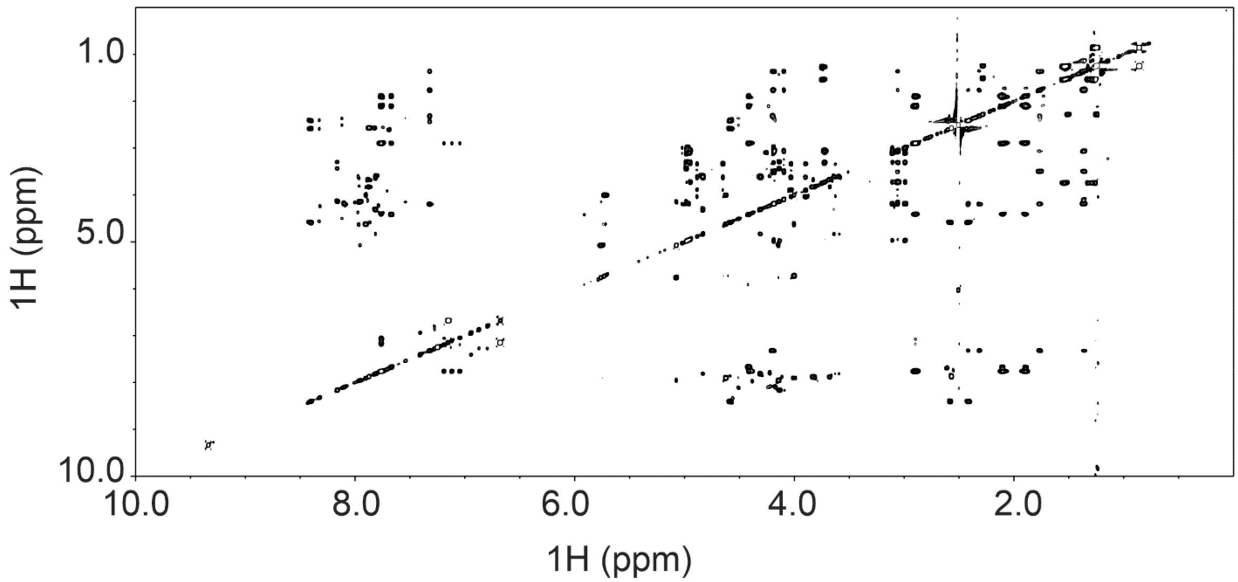

Figure S14. 2D TOCSY Spectra of N-methylamine-aspartic acid analogue (OCF-S3 and OCF-S4).

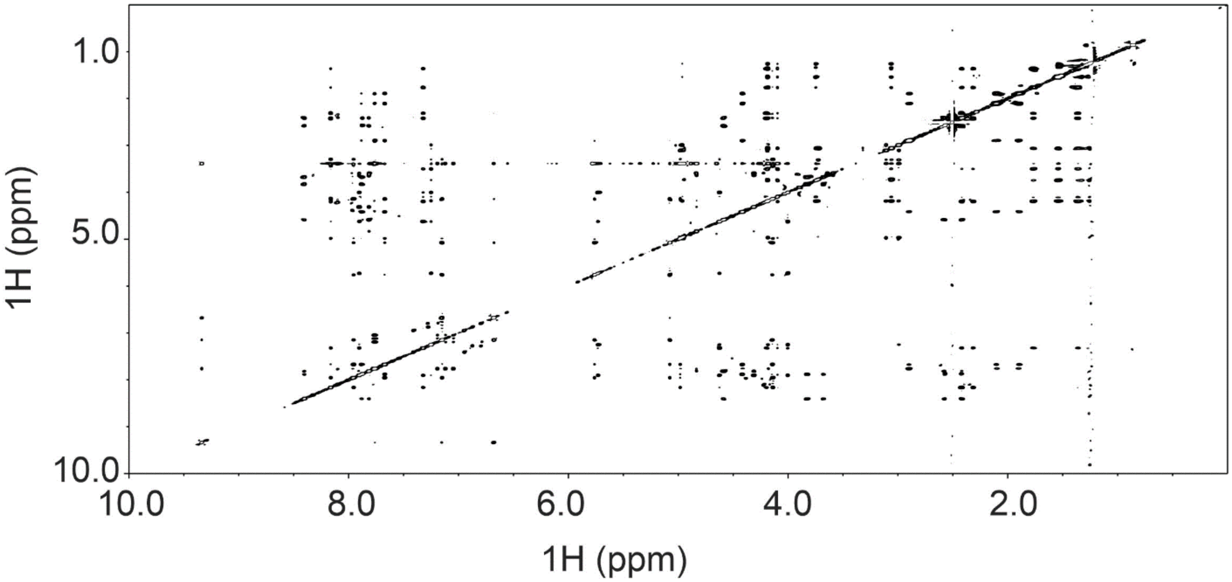

Figure S15. 2D NOESY Spectra of N-methylamine-aspartic acid analogue (OCF-S3 and OCF-S4).

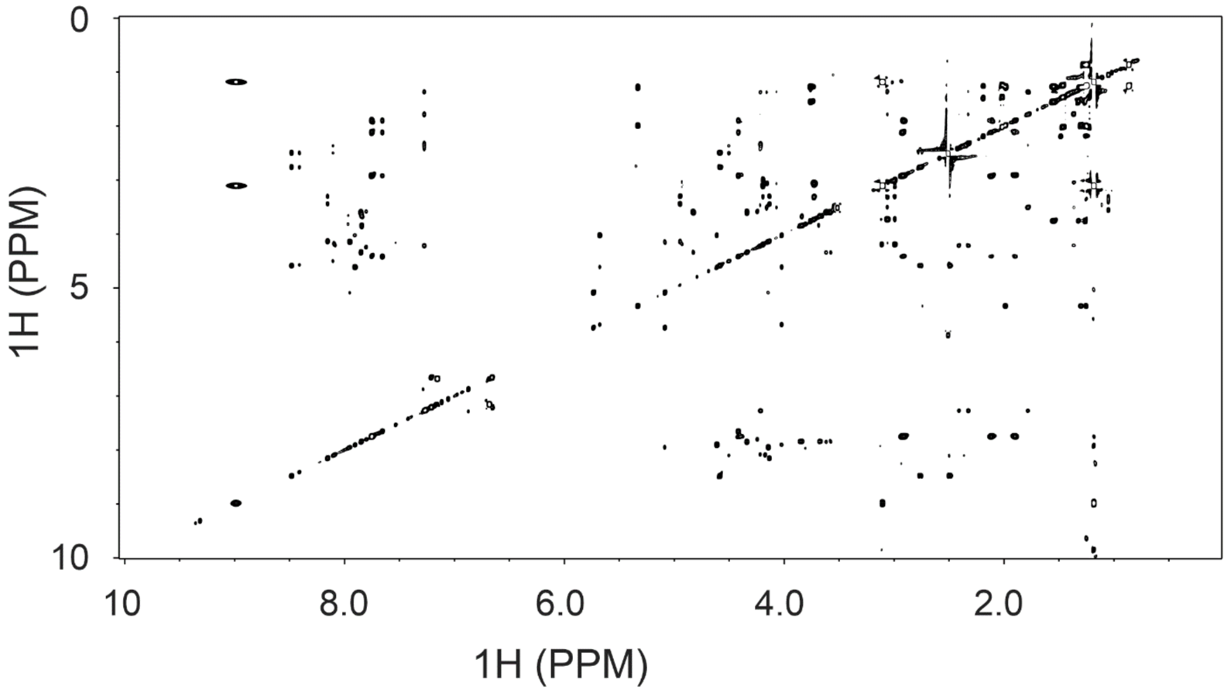

Figure S16. 2D TOCSY Spectra of ASP7 variants (OCF-E and OCF-F)

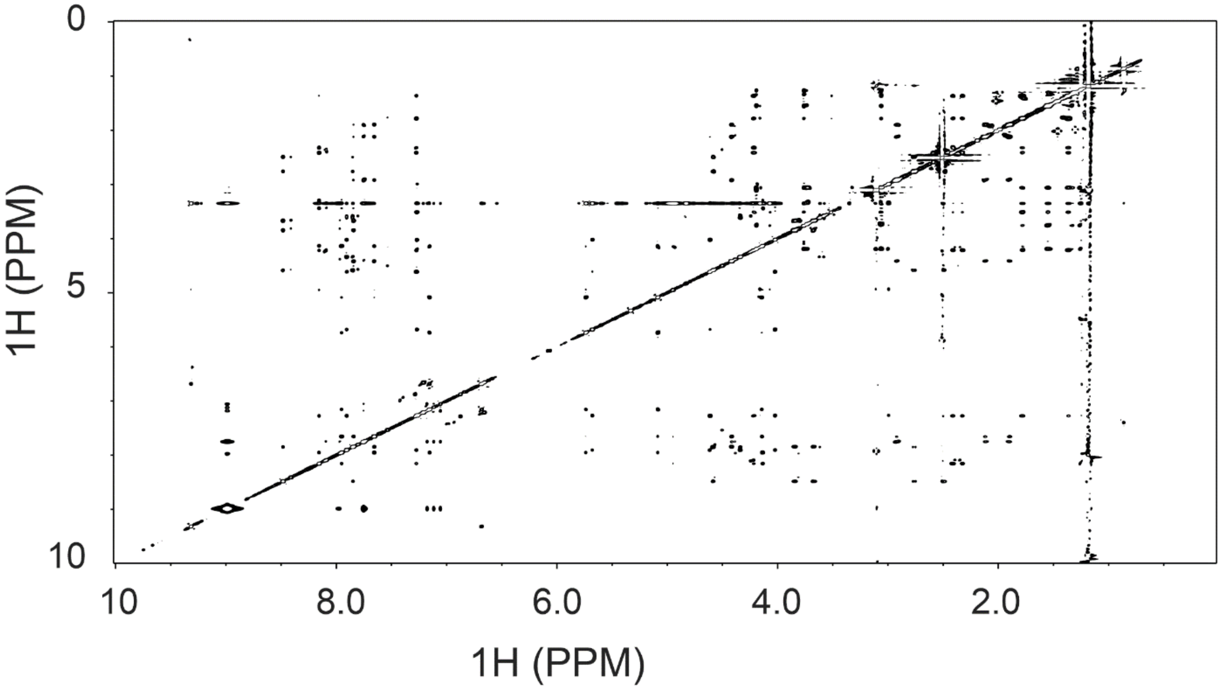

Figure S17. 2D NOESY Spectra of ASP7 variants (OCF-E and OCF-F)
